# Constipation

**Published:** 1876-07

**Authors:** 


"Why, you're as hearty as a buck!"

"Yes! I keep the bowels free, and read HALL'S JOURNAL OF HEALTH."

With the utmost confidence we advise all who are well, and desire to keep so, to follow the same prescription. As to those who are ailing, there can be no doubt that three fourths would get well of all ordinary maladies, by the same means; but the practical motto is: "Untold thousands to regain health; to retain it, not a dollar." Weeks and months of effort to arrest the progress of wasting disease; to prevent its commencement, not an hour. Such is the inconsideration, such the improvidence of nine persons out of ten, even among the educated.

"Continued care and anxiety of mind are also causes of constipation of the bowels. The great anxiety to which those are subjected who are continually and actively engaged in their business or profession, exerts a powerful influence upon the functions of the alimentary canal; it not only depresses the nervous system, and causes indigestion, but also renders torpid the peristaltic action of the bowels. As a confirmation of the truth of this, as soon as such persons emancipate themselves from the cares and anxieties of their business, by taking an excursion into the country, the mind soon recovers its wonted cheerfulness, the spirits their elasticity, and the bowels their normal function. Indeed, when we take into consideration the contentions, the competitions, and the responsibilities which this class of persons have daily to encounter, especially in large cities, it is a matter of no surprise that they should suffer from languor and depression, from indigestion and from constipation, with all its numerous evil consequences. To be forcibly impressed with the extent of the evils resulting from this cause, it is only necessary to pass along Broadway or the Bowery early in the morning, or late in the evening, and behold the multitude of anxious and careworn countenances which one sees daily at those hours in these two great thoroughfares of New-York. This spectacle will furnish a good commentary upon the *wear* and *tear* of human life.

